# BioPathfinder: Evidence-guided multi-agent platform enables hypothesis discovery for CAR-T engineering

**DOI:** 10.64898/2026.07.15.738646

**Published:** 2026-07-28

**Authors:** Shubing Wang, Yan-Ruide Li, Qian Wang, Youcheng Yang, Xinyuan Shen, Haoran Li, Haochen Nan, Zhoulin Chen, Yichen Zhu, Bo Zhang, Haoyan Ding, Jennifer Soto, Soomin Park, Yi Zheng, Xiangliang Huang, Debiao Li, Song Li

## Abstract

Clinical studies of chimeric antigen receptor (CAR)-T therapy generate diverse molecular and clinical evidence that remains fragmented across publications, public repositories and patient-derived datasets, limiting systematic therapeutic discovery. Here we present BioPathfinder, an evidence-guided multi-agent workflow for closed-loop biomedical discovery. BioPathfinder constructs a provenance-aware knowledge resource linking publications with patient single-cell RNA sequencing (scRNA-seq) datasets and clinical metadata, and uses role-specialized large language model agents to generate, review and prioritize diverse, falsifiable and dataset-aware mechanistic hypotheses for computational and experimental validation. Applied to a curated corpus of CAR-T-treated patient studies and matched scRNA-seq datasets, BioPathfinder identified candidate mechanisms underlying CAR-T persistence, dysfunction and therapeutic resistance. The workflow prioritized the hypothesis that genes associated with an NK-like transition programme could be targeted to reduce CAR-T exhaustion and improve persistence. Analysis of patient scRNA-seq datasets showed enrichment of this programme in exhausted post-infusion CAR-T cells. Virtual perturbation prioritized transition-associated receptor genes, including *KLRC1* , *KLRD1* and *KLRG1* , and expert review selected *KLRC1* , encoding NKG2A, for experimental validation. *In vitro* and *in vivo* chronic-stimulation models showed that NKG2A marked activated, exhaustion-associated CD8⁺ CAR-T cells, whereas NKG2A blockade enhanced antitumour activity and persistence-associated functional readouts *in vivo* . BioPathfinder establishes a generalizable framework that transforms fragmented clinical single-cell evidence into experimentally validated therapeutic hypotheses, providing a scalable strategy for AI-guided biomedical discovery.

**HIGHLIGHTS:**

1. BioPathfinder integrates fragmented CAR-T clinical studies, patient scRNA-seq datasets and metadata into a provenance-aware evidence resource for AI-guided hypothesis discovery.
2. A multi-agent workflow generates, critiques and prioritizes diverse, falsifiable, dataset-aware mechanisms underlying CAR-T persistence, dysfunction and therapeutic resistance.
3. BioPathfinder prioritizes KLRC1/NKG2A as a therapeutic target, and experimental validation demonstrates that NKG2A blockade enhances CAR-T persistence and antitumour function.

## Full Text Availability

The license terms selected by the author(s) for this preprint version do not permit archiving in PMC. The full text is available from the preprint server.

